# Atraumatic Subdural Hematoma in a Third-Trimester Gravid Patient

**DOI:** 10.1155/2016/8252746

**Published:** 2016-02-17

**Authors:** D. C. Traficante, A. Marin, A. Catapano

**Affiliations:** Department of Emergency Medicine, St. Joseph's Regional Medical Center, Paterson, NJ 07503, USA

## Abstract

Acute atraumatic subdural hematoma is a rare occurrence and there exist few case studies which describe suspected cases and causes for this condition. We present a case of a 36-year-old female at 32-week gestation who initially presented to the emergency department for evaluation of lower extremity cellulitis but had acute neurologic change while being in the ED. Computed tomography revealed a right subdural hematoma with midline shift and mass effect. The primary cause for the patient's subdural hematoma is unknown; however, this patient had several risk factors for developing an atraumatic subdural hematoma.

## 1. Introduction 

Acute atraumatic, or nontraumatic, subdural hematoma is a rare condition and much of the literature regarding this pathology is limited to case reports. Some described causes of atraumatic SDH include ruptured aneurysm, arteriovenous malformations, preeclampsia, cocaine abuse, and severe coagulopathy. The authors present a case of a third-trimester gravid patient with an acute atraumatic subdural hematoma.

## 2. Case Report 

A 36-year-old female G_6_P_2032_ at 32-week gestation presented to the emergency department for evaluation of bilateral lower extremity rash and leg pain. The patient reported that she was recently hospitalized for similar symptoms. Chart review showed that the patient was admitted to the hospital 8 weeks priorly for endocarditis, severe sepsis, and lower extremity cellulitis; however, she signed out against medical advice after approximately 2 weeks of treatment. The patient stated that she was noncompliant with her prenatal care and with the antibiotics prescribed to her when she signed out AMA approximately 6 weeks priorly. Past medical history was significant for Hepatitis C, IV drug abuse, and chronic lower extremity cellulitis. She admitted injecting heroin and cocaine into her right antecubital fossa prior to arrival in the hospital.

Physical exam on arrival revealed blood pressure of 103/53, pulse 117, respiratory rate 16, temperature 98.6 degrees Fahrenheit, and oxygen saturation of 97% on room air. The patient appeared older than her stated age, gravid, unkempt, and obviously ill. Her pupils were equal, round, and reactive to light and extraocular muscles were intact. Oral mucosa was dry, with stomatitis. Systolic 3/6 heart murmur was present, and she was tachycardic at a rate of ~120 bpm. She was alert and oriented to person, place, time, and situation; GCS was 15. In addition, she followed commands but was somewhat slow to respond. Skin exam revealed Janeway lesions and Osler nodes on the right and left hand. There were also bilateral lower extremity swelling and well demarcated erythema extending from the ankles to midcalf with scattered superficial ulcerations. There were no obvious outward signs of trauma.

Laboratories were obtained, along with three sets of blood cultures. Labs returned with WBC of 7.4, but with moderate bandemia of 23 bands. Hgb and Hct were 9.0 and 26.5, respectively. Platelets were 71,000. CMP revealed CO_2_ of 16, Cr of 1.74, and mildly elevated liver function tests. Urinalysis was grossly positive with large leukocyte esterase, too many WBC, and proteinuria >100. A troponin-I was obtained as patient had been complaining of chest pain, which returned to be elevated at 0.392. Chest X-ray was unremarkable. EKG showed sinus tachycardia without ischemic changes. A bedside ultrasound showed a positive intrauterine pregnancy consistent with 32-week gestation, positive fetal movement, and a fetal heart rate of 142 bpm.

**Figure 1:**
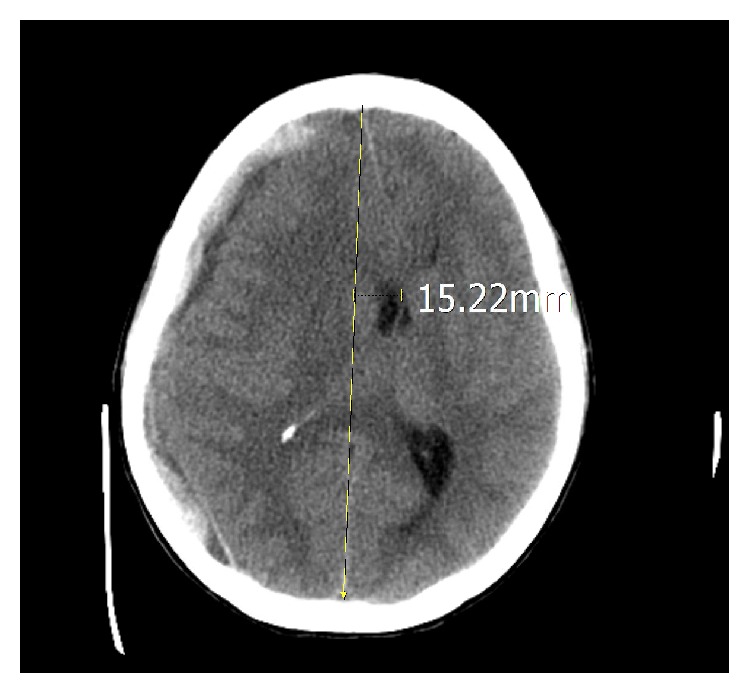
Computed tomography axial image showing right subdural hematoma with midline shift.

**Figure 2:**
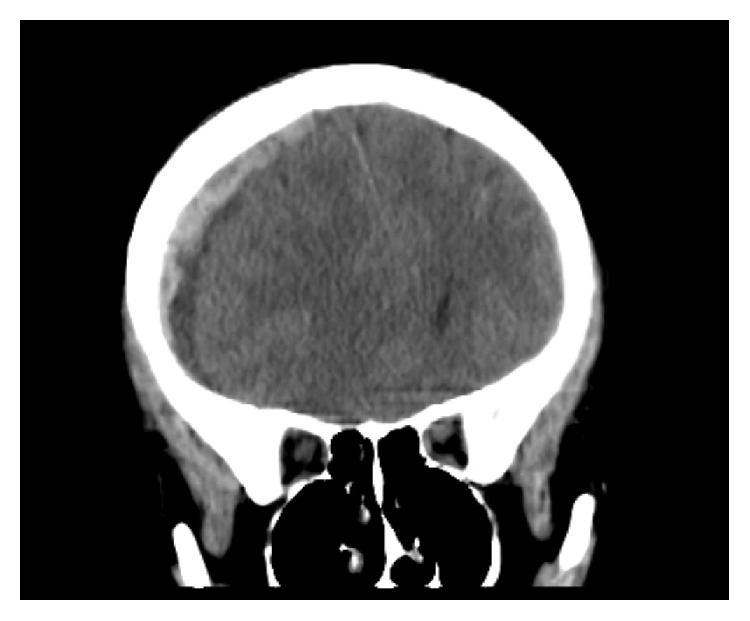
Computed tomography coronal image showing right subdural hematoma with midline shift.

The patient was started on a course of IV antibiotics and received 2L NS during the ED course. After approximately two hours in the department, the patient developed an acute change in mental status. She was less responsive, slurring her words, and found to have a right sided blown pupil. A stat CT scan revealed an acute right subdural hematoma with midline shift and mass effect (Figures 1 and 2). During the CT scan, the patient developed apnea and, therefore, was intubated for airway protection. She was given a bolus of mannitol and emergent consults were placed with neurosurgery and OB/GYN. She was immediately transferred to the operating room where she underwent simultaneous emergent cesarean section and right hemicraniectomy.

The patient's family decided to withdraw care and she died on day 3 in the surgical ICU, likely secondary to extensive cerebral infarcts and herniation. The patient's baby girl was discharged from the hospital into child protection custody on day 21.

## 3. Discussion 

A subdural hematoma is a collection of blood between the dura and arachnoid membrane layers of the meninges. It is the most common type of intracranial mass lesion, seen in approximately 5–25% of patients with severe head injuries, and is associated with high morbidity and mortality rates [1]. The most common cause of acute subdural hematoma is trauma. Causes of atraumatic subdural hematoma, which are much less common, include aneurysm rupture, ruptured cortical artery, hypertensive cerebral hemorrhage, arteriovenous malformations and dural arteriovenous fistula, neoplasms, hematological disorders, anticoagulant and thrombolytic therapy, cerebral amyloid angiopathy, acquired immunodeficiency syndrome, cocaine abuse, moyamoya disease, preeclampsia, HELLP syndrome, and severe coagulopathy. In addition, the pregnant patient undergoes physiological hormonally mediated changes in circulation, vascular tissue structure, and coagulability, all of which can contribute to further increased risk of stroke and bleeds [2, 3].

Prognosis is typically poor for patients with subdural hematomas, with the amount of associated direct brain damage and the damage resulting from the mass effect of the hematoma dictating the ultimate outcome. Mortality rates range from 16% to over 60% in some studies, with certain prognostic factors increasing the rate. These factors include increased age, time from injury to treatment, GCS on admission, immediate coma or lucid interval, CT findings (hematoma volume, degree of midline shift, associated intradural lesion, and compression of basal cisterns), postoperative ICP, and the type of surgery the patient underwent [4, 5]. Also several studies have shown increased mortality in patients with the presence of pupillary abnormalities, which was seen acutely in our patient. Koç et al. reported that patients with acute SDH who presented with bilateral or unilateral nonreactive pupils had mortality rates of 97% and 81%, respectively [6].

In our case, likely the combination of thrombocytopenia, preeclampsia, early HELLP syndrome, cocaine use, and disseminated intravascular coagulation secondary to severe sepsis may have all contributed to some extent to the development of an acute atraumatic subdural hematoma. Alternative causes in our patient's case could also include showered septic emboli secondary to her known recent history of endocarditis or ruptured mycotic aneurysm.

## 4. Conclusion 

Subdural hematomas are a relatively common finding in patients who present with severe head trauma. When there are no outward signs of trauma but there is a focal neurological sign or change, it is important to consider atraumatic subdural hematoma as a potential diagnosis. In our case, there was likely a multifactorial cause that ultimately resulted in the acute subdural hematoma with thrombocytopenia, early HELLP syndrome, sepsis, cocaine use, and possible preeclampsia all contributing to her condition. Acute subdural hematomas carry significant mortality in the pregnant patient. As this situation affects two patients, mother and fetus, care must be taken to detect these early and treat them effectively. In cases where the pregnant patient is unstable, it is important to weigh the risk of fetal compromise and to act quickly to deliver the baby if needed.
